# Social Risk and Clinical Outcomes Among Adults With Type 2 Diabetes

**DOI:** 10.1001/jamanetworkopen.2024.25996

**Published:** 2024-08-29

**Authors:** Rebekah J. Walker, Joni S. Williams, Sebastian Linde, Leonard E. Egede

**Affiliations:** 1 Division of Population Health, Department of Medicine, Jacobs School of Medicine and Biomedical Sciences, University at Buffalo, Buffalo, New York; 2 Division of General Internal Medicine, Department of Medicine, Medical College of Wisconsin, Milwaukee; 3 Center for Advancing Population Science, Medical College of Wisconsin, Milwaukee; 4 Department of Health Policy & Management, Texas A&M School of Public Health, College Station

## Abstract

**Importance:**

No clear process exists for categorizing social risks in a way that informs effective social risk screening and intervention development.

**Objective:**

To investigate social risk profiles and associations of those profiles with clinical outcomes in adults with diabetes using latent profile analysis.

**Design, Setting, and Participants:**

For this cross-sectional study, a latent profile analysis was conducted using data for adults with type 2 diabetes collected at 2 primary care clinics in the Southeastern US from 2013 to 2014. Data were analyzed from November to December 2023.

**Main Outcomes and Measures:**

Participants completed validated questionnaires for 26 social risk factors within 5 domains of social determinants of health: socioeconomic, neighborhood, education, food, and social and community context. In addition, participants completed questions that assessed psychological risk and behavioral risk. A 3-step latent profile analysis was used to identify different subgroups within the sample. Profiles were then regressed on outcomes of hemoglobin A_1c_ (HbA_1c_), blood pressure, and quality of life.

**Results:**

A total of 615 participants (mean [SD] age, 61.3 [10.9] years; 379 men [61.6%]) were included. Five latent class profiles were identified. The lowest risk group had significantly higher mental health–related quality of life compared with a group with higher neighborhood risk (β, 1.11; 95% CI, 0.67 to 1.55). The second group had low economic risk but high neighborhood risk and served as the reference group. The third group had high economic and neighborhood risk and had significantly higher blood pressure (β, 8.08; 95% CI, 2.16 to 14.01) compared with the reference. The fourth group had high psychological and behavioral risks but low socioeconomic and neighborhood risks. This group had significantly higher HbA_1c_ (β, 0.47; 95% CI, 0.01 to 0.92) and lower mental health–related quality of life (β, −1.83; 95% CI, −2.41 to −1.24) compared with the reference. The highest risk group indicated high risk in all domains, had significantly higher HbA_1c_ (β, 1.07; 95% CI, 0.50 to 1.63), and had lower mental health–related quality of life (β, −2.15; 95% CI, −2.87 to −1.42) compared with the reference.

**Conclusions and Relevance:**

These findings suggest that social risk profiles can be identified according to social, psychological, and behavioral risk domains and the health outcome of concern among adults with diabetes. Future work should consider the use of social risk profiles in intervention development and testing.

## Key Points

**Question:**

Are there social risk profiles associated with clinical outcomes in adults with type 2 diabetes?

**Findings:**

In a cross-sectional study using latent profile analysis for 615 adults with type 2 diabetes, 5 latent class profiles were identified. Groups were differentiated by risk of economic, neighborhood, psychological, and behavioral risks with differences in glycemic control, blood pressure, and quality of life.

**Meaning:**

These findings suggest that social risk profiles can be identified according to social, psychological, and behavioral risk domains in adults with diabetes and that such profiles could be used to identify ideal interventions for different risk groups.

## Teaser

This cross-sectional study investigates social risk profiles and associations of those profiles with clinical outcomes in adults with type 2 diabetes using latent profile analysis.

## Introduction

Diabetes is the seventh leading cause of death in the US and is associated with high morbidity, mortality, and health care costs.^1^ Approximately 95% of individuals with diabetes have type 2 diabetes, requiring substantial behavior change managed primarily by the individual with support from their health care practitioners.^1^ Individuals with type 2 diabetes who are nonsmokers and meet clinical goals for hemoglobin A_1c_ (HbA_1c_), blood pressure, and cholesterol are less likely to experience complications and increased mortality.^1,2^ However, only 11% of the US population meet the ideal criteria, and 36% meet less stringent criteria, highlighting the importance of addressing factors that limit individuals’ ability to manage diabetes.^1^

Social determinants of health are conditions in which people are born, grow, live, work, and age and that shape their health over the life course.^3,4^ These factors have been noted to account for between 30% to 55% of health outcomes, and are shaped by upstream factors, as well as adverse social conditions, commonly referred to as social risk.^3,5^ Extensive evidence highlights the role social determinants play in diabetes outcomes, including increased risk for those who are food insecure, have limited social support systems, live in neighborhoods with high crime and violence, report transportation or housing instability, and have poor health care access.^6,7,8,9,10^

Evidence suggests that combining medical care with interventions that target these social risk factors can improve health outcomes.^11,12^ However, there is limited information on how to group social risks in a way that can inform effective identification of interventions. A commonly accepted categorization of social determinants is the Kaiser Family Foundation framework, which groups these factors into (1) economic stability, (2) neighborhood and built environment, (3) education, (4) food, (5) social and community context, and (6) the health care system.^7,13^ This groups risk into conceptually similar categories, but there is limited work on whether combinations of different risks are important to target concurrently, as opposed to addressing each risk separately. Studies^14,15^ of national data found associations between a cumulative number of social risks and likelihood of diabetes, as well as likelihood of cardiovascular death. A study^16^ of individuals with type 2 diabetes participating in a trial addressing social needs also found a dose-response association between number of social risks and depression and anxiety. However, increasing numbers of social risk factors were not statistically significantly associated with glycemic control, and the magnitude of the association between social risk and cost-related nonadherence remained similar despite increasing number of risks.^16^ Therefore, a simple count of social risks may not be the best way to identify groups. In addition, the type of social risk is not considered in a simple count, which limits the ability to inform intervention development.

A method that is particularly useful in identifying subgroups with common characteristics that could benefit from a similar intervention is latent class analysis (LCA).^17^ Therefore, the aim of this study was to investigate social risk profiles and the association of those profiles with clinical outcomes in adults with diabetes using LCA.

## Methods

### Sample Population

Cross-sectional data from adults with type 2 diabetes were used in this analysis. All study procedures were approved by the Medical University of South Carolina institutional review board before initiation. Participants were recruited from 2 primary care clinics in the Southeastern US using posted flyers and mailed invitation letters. The study team posted flyers in clinics at the local academic medical center and the Veterans Affairs medical center, directing interested individuals to call the study line for more information. In addition, the study team sent invitation letters to adults with diabetes who had a primary care clinic visit during the prior year. Letters provided a brief introduction to the study goals and inclusion criteria with the same study line to call for additional information if interested. During conversations with interested individuals, study staff outlined the study goal, asked questions regarding inclusion criteria, confirmed that participants were willing to have staff access their medical records for laboratory values, and scheduled a visit time. Inclusion criteria included (1) age 18 years or older, (2) clinical diagnosis of type 2 diabetes confirmed in the medical record, and (3) ability to communicate in English as indicated by the participant. Exclusion criteria included mental confusion at the time of interview suggesting major dementia, alcohol and/or drug dependency, or active psychosis and/or acute mental disorder as determined by the study team member during the consenting process. Eligible participants gave written informed and completed an assessment that includes a suite of validated scales. Most recent glycemic control (HbA_1c_) and blood pressure measures were abstracted from the medical record following the study visit. Race and ethnicity were self-identified by the participants and are included to provide information on the demographic make-up of the study sample. Participants were given $25 for participation. This study follows the Strengthening the Reporting of Observational Studies in Epidemiology (STROBE) reporting guidelines for cross-sectional studies.

### Measures

A total of 26 measures were included in the analysis: 19 social risk factors, 5 psychological risk factors, and 2 behavioral risk factors. Validated scales were used to collect data on each measure. Each risk factor was treated as a continuous variable in the analysis.

The Kaiser Family Foundation Social Determinants of Health framework was used to categorize the 19 social risk factors.^13^ First, economic instability (3 measures) included (1) work hours, (2) income, and (3) subjective social status. Work hours and income were collected using questions previously validated from the 2002 National Health Interview Survey.^18^ Subjective social status is a perceived measure of socioeconomic status where respondents mark on a ladder with 10 rungs which rung they would place themselves if 10 are the people with the most money, education, and well-respected job, and 1 are the people with the least money, education, and well-respected jobs.^19^

Second, neighborhood and built environment (10 measures) included (1) neighborhood aesthetics, (2) neighborhood walkability, (3) neighborhood safety, (4) violence, (5) crime, (6) neighborhood rating, (7) neighborhood comparability, (8) recreational facilities, (9) neighborhood activities, and (10) neighborhood problems. Measures were captured using questions from validated neighborhood characteristics scales and indices published by Echeverria et al.^20^ Neighborhood comparability asked how their neighborhood compared to others in their county.^20^ Recreational facilities index asked about the presence of recreational facilities and quality of facilities.^20^ Neighborhood activities questions asked about participation in civic and political activities with neighbors.^20^ Neighborhood problems asked about whether certain characteristics, such as presence of trash and litter, were a problem.^20^

Third, education (2 measures) included (1) education level and (2) health literacy. Education was captured using a measure of the number of years of education from the 2002 National Health Interview Survey.^18^ Health literacy was captured using the abbreviated version of the Test of Functional Health Literacy in Adults scale.^21^

Fourth, food environment (2 measures) included (1) food insecurity and (2) food access. Food insecurity was captured using questions from the US Department of Agriculture 6-item food insecurity scale.^22^ Food access was captured using questions from the 11-item access to healthy food scale in the neighborhood characteristics scales published by Echeverria et al.^20^

Fifth, social and community context (2 measures) included (1) social isolation and (2) discrimination. Social isolation was captured using questions about social cohesion from neighborhood characteristics scales published by Echeverria et al.^20^ Discrimination was captured using previously validated items on discrimination over the past 12 months according to race and ethnicity, level of education, sex or gender, or language from the DISTANCE survey.^23^

In addition, validated scales for psychological risk factors known to influence diabetes outcomes and be associated with social risk were created. Sixth, psychological risk factors (5 measures) included (1) diabetes fatalism measured using the 12-item Diabetes Fatalism Scale,^24^ (2) depression measured using the 9-item Patient Health Questionnaire–9,^25^ (3) diabetes distress measured using the 17-item Diabetes Distress Scale,^26^ (4) serious psychological distress measured using the 6-item Kessler-6 scale developed for nonspecific psychological distress,^27^ and (5) and perceived stress measured using the 4-item Perceived Stress Scale.^28^

Seventh, behavioral risk factors (2 measures) included (1) substance use and (2) smoking. Substance use was measured using the 4-item CAGE (Cutting Down, Annoyance by Criticism, Guilty Feeling, and Eye-Openers)–Adapted to Include Drug screening instrument.^29^ Smoking was measured using questions previously validated from the 2002 National Health Interview Survey.^18^

### Outcome Measures

Three outcome measures were investigated to understand the association between social risk profiles and diabetes outcomes. First, for glycemic control, the most recent HbA_1c_ measure was abstracted from the medical record and treated as a continuous measure. Second, for blood pressure, the most recent systolic and diastolic blood pressure was abstracted from ambulatory visits in the medical record, with systolic blood pressure treated as a continuous measure in this analysis. Third, for quality of life, the valid and reliable Short Form Health Survey—12^30^ was used as a general measure of health-related quality of life. Questions were scored following published guidelines with summary physical health (Physical Component Scale–12) and mental health (Mental Component Scale–12) scores treated as separate continuous measures.

### Statistical Analysis

Data were analyzed from November to December 2023. LCA is a statistical process used to identify latent heterogeneity in a sample and create subgroups, termed latent classes, based on these characteristics.^17,31^ This method can be used with categorical or continuous data; when used with continuous data, it is termed *latent profile analysis*.^17^ Individual responses on a set of measures are investigated for patterns in responses that would differentiate subgroups or determine class membership.^17^ Conceptually similar to cluster analysis, LCA generates probabilities of class membership, which are assessed through statistical and theoretical criteria.^17^ According to a recent review of best practices for LCA,^17^ a sample size of 300 or more cases is desirable, so this study has sufficient power to conduct LCAs.

Sample demographics and risk factors were summarized using descriptive statistics. Three-step latent profile analysis in Stata statistical software version 17 (StataCorp) was then used to identify subgroups of social risk within the dataset. Following best practice procedures, a sequence of models starting with a lower number of possible classes and specifying 1 additional class at a time, were then run, followed by comparison on statistical criteria.^17^ Two-class, 3-class, 4-class, and 5-class profiles were investigated. Each model was then assessed using Akaike Information Criterion (AIC) and Bayesian Information Criterion (BIC) scores, where lower scores indicate better fit. After selecting the class profile with the optimal number of classes, we used posterior probability to classify individuals into their true latent class. Accuracy of classification was determined by the entropy score. Entropy values close to 1 indicate models that accurately define classes, with scores above 0.8 considered acceptable and scores above 0.9 considered ideal.^17^ Marginal means for each measure were investigated across the different latent classes, in addition to proportions of the sample included in each group.

Finally, to validate the selected class solution, profiles were tested against outcomes of glycemic control, blood pressure, and quality of life (physical and mental components). Linear regression was used for each of the 4 continuous variable outcomes with the 5 classes treated as a categorical primary independent variable. Coefficients can be interpreted as the change in the outcome for a specified class compared with the reference class. Statistical significance was assessed using 2-sided *P* < .05.

## Results

Table 1 provides demographic characteristics of the 615 participants (mean [SD] age, 61.3 [10.9] years; 379 men [61.6%]; 236 women [38.4%]) included in the study. Most of the sample (399 individuals [64.9%]) was African American or Black, 203 (33.0%) were White, and 13 (2.1%) were another race or ethnicity (eg, American Indian or Alaska Native, Asian, or multiracial). Nearly one-half of the sample (247 individuals [41.6%]) had an income less than $20 000 per year.

**Table 1.** Demographic Characteristics of Adults With Type 2 Diabetes Included in Study

Characteristic	Participants, No. (%) (N = 615)
Age, mean (SD), y	61.3 (10.9)
Diabetes duration, mean (SD), y	12.3 (9.1)
Sex	
Female	236 (38.4)
Male	379 (61.6)
Self-reported race	
African American or Black	399 (64.9)
White	203 (33.0)
Other race or ethnicity^a^	13 (2.1)
Marital status	
Never married	69 (11.2)
Married	305 (49.7)
Separated or divorced	173 (28.2)
Widowed	67 (10.9)
Annual income, $	
<20 000	247 (41.6)
20 000-34 999	149 (25.1)
35 000-49 999	82 (13.8)
50 000-74 999	60 (10.1)
≥75 000	56 (9.4)
Education, mean (SD), y	13.4 (2.8)
Employment, mean (SD), h/wk	12.5 (19)
Insurance	
None	57 (9.3)
Private	124 (20.2)
Medicare	152 (24.7)
Medicaid	63 (10.2)
Veterans Affairs	147 (23.9)
Other	72 (11.7)
Charlson Comorbidity Index score, mean (SD)	25.7 (2.2)
Health status	
Excellent or very good	82 (13.3)
Good	235 (38.2)
Fair	238 (38.7)
Poor	60 (9.8)

^a^ Other race or ethnicity includes American Indian or Alaska Native, Asian, and multiracial.

Table 2 provides a summary of responses to the social, psychological, and behavioral risk variables organized by category, with the mean (SD), minimum, and maximum for each score. The 5-class profile was identified as the best fit profile on the basis of investigation of AIC and BIC for each of the tested models. The 2-class model had an AIC of 74 700.58 and BIC of 75 049.89. The 3-class model had an AIC of 74 051.65 and BIC of 74 520.35. The 4-class model had an AIC of 73 589.19 and BIC of 74 177.27. The 5-class model had an AIC of 73 305.25 and BIC of 74 012.71. Entropy score for the 5-class profile was 0.99, indicating high accuracy of classification.

**Table 2.** Social Risk Latent Class Variable Indicators

Social risk category and social risk variable	Score, mean (SD) [range]
Economic instability	
Work hours	12.5 (19.0) [0-80]
Income category	3.2 (2.3) [0-7]
Subjective social status	6.1 (2.4) [1-11]
Neighborhood and built environment	
Neighborhood aesthetics	15.2 (2.8) [5-25]
Neighborhood walkability	28.6 (7.1) [11-55]
Neighborhood safety	8.5 (2.0) [3-15]
Violence	5.1 (2.0) [4-16]
Crime	2.1 (0.8) [1-4]
Neighborhood rating	1.8 (0.8) [1-4]
Neighborhood comparability	2.1 (0.9) [1-5]
Recreational facilities	4.4 (2.5) [0-8]
Neighborhood activities	8.9 (3.2) [0-12]
Neighborhood problems	12.4 (3.7) [0-16]
Education access and quality	
Years of education	13.4 (2.8) [1-24]
Health literacy	26.1 (10.2) [0-36]
Food risks	
Food insecurity	1.3 (1.9) [0-6]
Food access	16.2 (7.1) [6-30]
Social and community context	
Social isolation	12.6 (3.4) [5-25]
Discrimination	4.7 (1.7) [4-15]
Psychological risks	
Fatalism	34.0 (9.5) [12-71]
Depression	6.1 (6.0) [0-27]
Diabetes distress	1.6 (0.7) [1-5.3]
Psychological distress	5.8 (6.3) [0-24]
Perceived stress	5.3 (3.3) [0-15]
Behavioral risks	
Alcohol and/or drug use	0.5 (1.0) [0-4]
Smoking	1.7 (0.8) [1-3]

Table 3 provides expected proportions of the sample within each of the 5 latent class profiles and marginal means and probabilities for each measure in each of the 5 latent class profiles. According to the characteristics of the 5 classes, group 1 had the lowest overall risk. Group 2 had low economic risk, but high neighborhood risk. Group 3 had high economic and neighborhood risk. Group 4 had high psychological and behavioral risk and had significantly higher HbA_1c_ (β, 0.47; 95% CI, 0.01 to 0.92) and lower mental health–related quality of life. Group 5 had the highest overall risk, with high risk in the economic, neighborhood, psychological, and behavioral risk categories; this group had significantly higher HbA_1c_ (β, 1.07; 95% CI, 0.50 to 1.63) and lower mental health–related quality of life. Group 1 (36%) and group 2 (34%) had the highest proportions of the sample, with group 3 (6%) and group 5 (8%) having the lowest proportions. Group 4 accounted for 14% of the sample. Characteristics of the social risk profiles are shown in Table 4.

**Table 3.** Five Latent Class Profiles

Risk factor and variables	Marginal means for each measure				
	Group 1	Group 2	Group 3	Group 4	Group 5
Percentage of sample	36	34	6	14	8
Economic risk					
Work hours	15.6	12.6	6.7	9.4	8.2
Income	4.2	3.1	1.4	2.3	1.7
Subjective social status	6.9	5.9	6.5	4.9	4.5
Years of education	14.2	13.3	12.2	13.0	12.1
Health literacy	28.3	26.2	17.1	24.3	26.4
Neighborhood risk					
Aesthetics	14.3	16.3	14.7	14.6	16.5
Walkability	24.7	32.1	28.9	26.7	35.1
Safety	7.5	9.2	8.0	8.5	10.5
Violence	4.3	4.8	9.5	4.9	7.8
Crime	1.5	2.3	3.0	2.0	3.1
Neighborhood rating	1.2	2.0	2.8	1.7	2.9
Comparability	1.5	2.3	2.9	2.0	3.2
Recreational facilities	3.4	5.5	3.8	3.7	6.3
Neighborhood activities	7.9	9.9	7.2	8.6	10.3
Neighborhood problems	14.8	12.0	6.2	13.4	6.6
Food insecurity	0.5	1.1	2.1	2.5	3.4
Food access	12.0	19.7	15.5	15.5	22.1
Social isolation	10.2	13.7	15.3	12.8	16.4
Discrimination	4.3	4.5	5.1	5.2	6.9
Psychological risk					
Fatalism	31.9	33.5	33.2	37.5	40.5
Depression	3.0	4.5	5.3	13.0	15.1
Diabetes distress	1.3	1.4	1.4	2.2	2.9
Psychological distress	3.2	4.4	6.3	11.1	13.1
Perceived stress	3.8	4.9	5.5	8.0	8.8
Behavioral risk					
Alcohol/drug use	0.3	0.3	0.4	1.5	0.8
Smoking	1.7	1.6	1.7	2.0	2.0

**Table 4.** Characteristics of Social Risk Profiles

Risk variable	Group 1	Group 2	Group 3	Group 4	Group 5
Economic risk	Low risk	Low risk	High risk	Average risk	High risk
Neighborhood risk	Average risk	High risk	High risk	Average risk	High risk
Psychological risk	Average risk	Average risk	Average risk	High risk	High risk
Behavioral risk	Average risk	Average risk	Average risk	High risk	High risk

Table 5 provides β coefficients for the association of each class with diabetes outcomes. Group 2, which had low economic risks but high neighborhood social risks, was used as the reference group. On the basis of prior research,^14,15,16^ individuals with low risk would be expected to show differences from those with higher risk. Therefore, a group with some risk was chosen as the reference to better indicate whether the latent profiles could differentiate risk. Compared with this group, individuals in group 4 (β, 0.47; 95% CI, 0.01 to 0.92) and group 5 (β, 1.07; 95% CI, 0.50 to 1.63) had significantly higher HbA_1c_ (worse glycemic control) and significantly worse mental health–related quality of life (group 4, β, −1.83; 95% CI, −2.41 to −1.24; group 5, β, −2.15; 95% CI, −2.87 to −1.42). Compared with group 2, individuals in group 3 (high economic and neighborhood risk) had significantly higher blood pressure (β, 8.08; 95% CI, 2.16 to 14.01). In addition, individuals in group 1 (overall low risk) had significantly higher mental health–related quality of life (β, 1.11; 95% CI, 0.67 to 1.55). There were no statistically significant differences in physical health–related quality of life.

**Table 5.** Diabetes Outcomes by Class Membership

Group	β (95% CI) for linear regression models			
	Hemoglobin A_1c_	Blood pressure	Physical health quality of life	Mental health quality of life
Group 2	0 [Reference]	0 [Reference]	0 [Reference]	0 [Reference]
Group 1 (overall low risk)	−0.21 (−0.55 to 0.13)	2.33 (−0.79 to 5.46)	−0.06 (−0.24 to 0.12)	1.12 (0.67 to 1.55)^a^
Group 3 (economic and neighborhood risk)	0.42 (−0.23 to 1.08)	8.10 (2.16 to 14.01)^b^	−0.16 (−0.50 to 0.18)	−0.23 (−0.05 to 0.60)
Group 4 (psychological and behavioral risk)	0.47 (0.01 to 0.92)^c^	−0.81 (−4.97 to 3.35)	0.05 (−0.19 to 0.30)	−1.83 (−2.41 to −1.24)^a^
Group 5 (high risk in all categories)	1.07 (0.50 to 1.63)^a^	1.30 (−3.91 to 6.48)	−0.07 (−0.37 to 0.23)	−2.15 (−2.87 to −1.42)^a^

^a^ *P* < .001.

^b^ *P* < .01.

^c^ *P* < .05.

## Discussion

In this cross-sectional study, using a sample of adults with type 2 diabetes, we identified 5 latent class profiles to guide development and implementation of interventions based on risk and outcome of interest. Group 1, the lowest risk group, accounted for 36% of the sample, and had limited risk but indicated some concerns about their neighborhood. This group had significantly higher mental health quality of life than those with higher neighborhood risk. Group 2 accounted for 34% of the sample, and, although overall their risk was low, they considered their neighborhoods to have low aesthetics, low food access, and high social isolation. Group 3 accounted for 6% of the sample and had high social risk in terms of neighborhood crime, violence, and financial instability, but low psychological and behavioral risk. This group had significantly higher blood pressure than those with lower economic risk. Group 4, which accounted for 14% of the sample, had high psychological and behavioral risk, but low socioeconomic and neighborhood risks. This group had significantly higher glycemic control (nearly 0.5%) and worse mental health–related quality of life compared with those with neighborhood risk. Finally, group 5, was the highest risk group, indicating high risk in all domains, and accounted for 8% of the sample. This group had significantly worse glycemic control (1% higher) and the lowest mental health–related quality of life.

This study adds to the limited research on latent profiles of social risk that can be used to guide intervention development and testing. A study^17^ of Black adolescents found differences in class membership by school safety and neighborhood safety. A second study^32^ of adults in India identified 3 clusters of cardiovascular disease risk: low risk, social risk, and behavioral risk. In the present study, we found differences in class membership by combinations of economic, neighborhood, psychological, and behavioral risks. Importantly, these risk groups matched differentially to important diabetes outcomes. Worse HbA_1c_ and lower mental health–related quality of life were associated with both groups that had high psychological and behavioral risk, with higher risk for those who also had economic and neighborhood risk. This finding supports the important role psychological health plays in outcomes for adults with diabetes^33^ but highlights that a compounded burden of economic and neighborhood risks in addition to psychological and behavioral risk may require a different approach to improve health outcomes. Specifically, interventions should incorporate strategies to target multiple risk factors simultaneously. Higher blood pressure was associated with the group with high economic and neighborhood risk but low psychological and behavioral risk. This suggests an important area of focus being blood pressure for individuals identifying high social risk without concurrent psychological or behavioral risk. A recent scientific statement from the American Heart Association^34^ noted the importance of improving psychological health to benefit cardiovascular health with recommendations for screening and interventions. Therefore, these findings highlight the importance of effective screening and interventions for individuals with high social risk beyond psychological risk.

An important implication of this work is the use of social risk profiles to identify interventions that will be most effective in improving health outcomes for specific subgroups of adults with type 2 diabetes. Rather than approaching individuals with a one-size-fits-all approach, these findings suggest interventions that target different domains of risk may need to be assessed with populations that identify these areas of risk as particularly problematic for them. Interventions may need to target multiple risk factors simultaneously, as opposed to tailoring for a single social risk factor or a single behavioral or psychological risk factor.

Findings from this study also highlight the importance of capturing different domains of risk in screening tools. In a review^35^ of social determinant screening in health care settings, health literacy was the most common risk screened for, followed by trauma history, social support, food insecurity, and housing. On the basis of the current results, ensuring screening for both economic and neighborhood risk, in addition to psychological and behavioral risks, may be important to identify populations at particularly high risk for poor outcomes. Selection of the tool and what domains to capture is an important area of discussion. A review^36^ of social risk screening tools in 2019 found more than 20 unique tools published at that time with no standard to follow. A common risk screening tool developed by the Centers for Medicare & Medicaid Services^37^ captures multiple risk types and, if used, could serve to identify risk in different domains. However, there are no questions related to an individual’s neighborhood, which is an important domain to capture according to the current analysis. A more recent review^38^ of screening tools found questions related to food insecurity and the physical environment in which a person lives are most frequently included, followed by economic stability and some aspect of social and community context. A focus on ensuring questions are asked across multiple risk types, instead of a focus on the number of risks screened, may help in selecting an appropriate tool and may help with feasibility of social risk screening. In a study^39^ investigating implementation of health-related social needs screening, individuals with fewer clinician visits and lower comorbidity burden were less likely to be screened. Therefore, the type of screening tool and the process for screening are important considerations in addition to which tool to use.^38^

### Limitations

Despite the novel use of LCA, there are limitations to this study worth noting. First, data were based on a cross-sectional sample, so no causality of effect should be inferred from these findings. Second, data were collected from adults with type 2 diabetes at 2 primary care clinics in the Southeastern US, so generalizability to other populations and regions should be tentative. Although we have no expectation that the influence of social risk on outcomes would differ, differentiation of risks into these profiles could be a unique aspect of a chronic disease, such as diabetes, access to health care through primary care clinics, and/or prevailing trends in a region of the nation. In addition, aspects of this sample may be unique, and samples with different race or ethnicity, sex, or income characteristics may identify different social risk profiles. Collection of data and testing of discrimination of social risk in different populations and regions will be important to validate these findings. In particular, future studies testing this method in healthy adults or individuals with different disease states will be informative for creation of a fully validated set of profiles. Furthermore, collection and inclusion of additional measures, such as more detailed information on housing or behavioral risk related to diet and physical activity, may result in different profiles and should be tested.

## Conclusions

In conclusion, this study aimed to identify social risk profiles that could guide social risk screening and intervention development to improve health for adults with type 2 diabetes. These findings suggest that social risk profiles can be created identifying individuals with differing levels of economic, neighborhood, psychological, and behavioral risk and that these profiles could be used to identify ideal interventions for different risk groups. This study also highlights the importance of capturing different domains of risk in screening tools. Future work should investigate differences in social risk profiles by disease state and region and should consider social risk profiles in intervention development and testing.

## Notes

Supplement.
Data Sharing Statement

## References

[1] Centers for Disease Control and Prevention (CDC). National diabetes statistics report. Accessed January 25, 2024. https://www.cdc.gov/diabetes/php/data-research/?CDC_AAref_Val=https://www.cdc.gov/diabetes/data/statistics-report/index.html

[2] American Diabetes Association Professional Practice Committee. Comprehensive medical evaluation and assessment of comorbidities: standards of care in diabetes-2024. Diabetes Care. 2024;47(suppl 1):S52-S76. doi:10.2337/dc24-S004 38078591 PMC10725809

[3] World Health Organization. Social determinants of health. Accessed January 25, 2024. https://www.who.int/health-topics/social-determinants-of-health#tab=tab_1

[4] World Health Organization. Closing the gap in a generation: health equity through action on the social determinants of health—final Report of the Commission on Social Determinants of Health. August 27, 2008. Accessed July 15, 2024. https://www.who.int/publications/i/item/WHO-IER-CSDH-08.1

[5] Alderwick H, Gottlieb LM. Meanings and misunderstandings: a social determinants of health lexicon for health care systems. Milbank Q. 2019;97(2):407-419. doi:10.1111/1468-0009.12390 31069864 PMC6554506

[6] Walker RJ, Smalls BL, Campbell JA, Strom Williams JL, Egede LE. Impact of social determinants of health on outcomes for type 2 diabetes: a systematic review. Endocrine. 2014;47(1):29-48. doi:10.1007/s12020-014-0195-0 24532079 PMC7029167

[7] Hill-Briggs F, Adler NE, Berkowitz SA, . Social determinants of health and diabetes: a scientific review. Diabetes Care. 2020;44(1):258-279. doi:10.2337/dci20-0053 33139407 PMC7783927

[8] Walker RJ, Garacci E, Palatnik A, Ozieh MN, Egede LE. The longitudinal influence of social determinants of health on glycemic control in elderly adults with diabetes. Diabetes Care. 2020;43(4):759-766. doi:10.2337/dc19-1586 32029639 PMC7085811

[9] Gold R, Kaufmann J, Gottlieb LM, . Cross-sectional associations: social risks and diabetes care quality, outcomes. Am J Prev Med. 2022;63(3):392-402. doi:10.1016/j.amepre.2022.03.011 35523696 PMC10286629

[10] Hill-Briggs F, Fitzpatrick SL. Overview of social determinants of health in the development of diabetes. Diabetes Care. 2023;46(9):1590-1598. doi:10.2337/dci23-0001 37354331

[11] Egede LE, Walker RJ, Linde S, . Nonmedical interventions for type 2 diabetes: evidence, actionable strategies, and policy opportunities. Health Aff (Millwood). 2022;41(7):963-970. doi:10.1377/hlthaff.2022.00236 35759702 PMC9563395

[12] Yan AF, Chen Z, Wang Y, . Effectiveness of social needs screening and interventions in clinical settings on utilization, cost, and clinical outcomes: a systematic review. Health Equity. 2022;6(1):454-475. doi:10.1089/heq.2022.0010 35801145 PMC9257553

[13] Artiga S, Hinton E. Beyond health care: the role of social determinants in promoting health and health equity. Kaiser Family Foundation. May 10, 2018. Accessed September 19, 2023. https://www.kff.org/racial-equity-and-health-policy/issue-brief/beyond-health-care-the-role-of-social-determinants-in-promoting-health-and-health-equity/

[14] Echouffo-Tcheugui JB, Caleyachetty R, Muennig PA, Narayan KM, Golden SH. Cumulative social risk and type 2 diabetes in US adults: the National Health and Nutrition Examination Survey (NHANES) 1999-2006. Eur J Prev Cardiol. 2016;23(12):1282-1288. doi:10.1177/2047487315627036 26763901

[15] Zhang W, Ahmad MI, Soliman EZ. The role of traditional risk factors in explaining the social disparities in cardiovascular death: the national health and Nutrition Examination Survey III (NHANES III). Am J Prev Cardiol. 2020;4:100094. doi:10.1016/j.ajpc.2020.100094 34327470 PMC8315458

[16] Leung CW, Heisler M, Patel MR. Multiple social risk factors are adversely associated with diabetes management and psychosocial outcomes among adults with diabetes. Prev Med Rep. 2022;29:101957. doi:10.1016/j.pmedr.2022.101957 36161137 PMC9502323

[17] Weller BE, Bowen NK, Faubert SJ. Latent class analysis: a guide to best practice. J Black Psychol. 2020;46(4):287-311. doi:10.1177/0095798420930932

[18] National Center for Health Statistics. Survey questionnaire, National Health Interview Survey, 2002. National Center for Health Statistics. 2004. Accessed September 19, 2023. https://www.cdc.gov/nchs/nhis/data-questionnaires-documentation.htm

[19] Cundiff JM, Smith TW, Uchino BN, Berg CA. Subjective social status: construct validity and associations with psychosocial vulnerability and self-rated health. Int J Behav Med. 2013;20(1):148-158. doi:10.1007/s12529-011-9206-1 22200973

[20] Echeverria SE, Diez-Roux AV, Link BG. Reliability of self-reported neighborhood characteristics. J Urban Health. 2004;81(4):682-701. doi:10.1093/jurban/jth151 15466849 PMC3455923

[21] Baker DW, Williams MV, Parker RM, Gazmararian JA, Nurss J. Development of a brief test to measure functional health literacy. Patient Educ Couns. 1999;38(1):33-42. doi:10.1016/S0738-3991(98)00116-5 14528569

[22] Bickel G, Nord M, Price C, Hamilton W, Cook J. Guide to measuring household food security (revised 2000). US Department of Agriculture, Food and Nutrition Service. March 2000. Accessed July 15, 2024. https://fns-prod.azureedge.us/sites/default/files/FSGuide.pdf

[23] Moffet HM, Adler N, Schillinger D, . Cohort profile: Diabetes Study of North California (DISTANCE)—objectives and design of a survey follow-up study of social health disparities in a managed care population. Int J Epidemiol. 2009;38(1):38-47. doi:10.1093/ije/dyn040 18326513 PMC2635421

[24] Egede LE, Ellis C. Development and psychometric properties of the 12-item diabetes fatalism scale. J Gen Intern Med. 2010;25(1):61-66. doi:10.1007/s11606-009-1168-5 19908102 PMC2811603

[25] Kroenke K, Spitzer RL, Williams JB. The PHQ-9: validity of a brief depression severity measure. J Gen Intern Med. 2001;16(9):606-613. doi:10.1046/j.1525-1497.2001.016009606.x 11556941 PMC1495268

[26] Fisher L, Glasgow RE, Mullan JT, Skaff MM, Polonsky WH. Development of a brief diabetes distress screening instrument. Ann Fam Med. 2008;6(3):246-252. doi:10.1370/afm.842 18474888 PMC2384991

[27] Kessler RC, Andrews G, Colpe LJ, . Short screening scales to monitor population prevalences and trends in non-specific psychological distress. Psychol Med. 2002;32(6):959-976. doi:10.1017/S0033291702006074 12214795

[28] Cohen S, Williamson G. Perceived stress in a probability sample of the United States. Spacapan S, Oskamp S, eds. The Social Psychology of Health. Sage; 1988:31-67.

[29] Ewing JA. Detecting alcoholism: the CAGE questionnaire. JAMA. 1984;252(14):1905-1907. doi:10.1001/jama.1984.03350140051025 6471323

[30] Ware J Jr, Kosinski M, Keller SDA. A 12-Item Short-Form Health Survey: construction of scales and preliminary tests of reliability and validity. Med Care. 1996;34(3):220-233. doi:10.1097/00005650-199603000-00003 8628042

[31] Hagenaars JA, McCutcheon AL, eds. Applied Latent Class Analysis. Cambridge University Press; 2002.

[32] Madavanakadu Devassy S, Webber M, Scaria L, . Social and behavioural risk factors in the prevention and management of cardiovascular disease in Kerala, India: a catchment area population survey. BMC Cardiovasc Disord. 2020;20(1):327. doi:10.1186/s12872-020-01595-x 32641078 PMC7346640

[33] American Diabetes Association Professional Practice Committee. Facilitating positive health behaviors and well-being to improve health outcomes: standards of care in diabetes—2024. Diabetes Care. 2024;47(suppl 1):S77-S110. doi:10.2337/dc24-S005 38078584 PMC10725816

[34] Levine GN, Cohen BE, Commodore-Mensah Y, . Psychological health, well-being, and the mind-heart-body connection: a scientific statement from the American Heart Association. Circulation. 2021;143(10):e763-e783. doi:10.1161/CIR.0000000000000947 33486973

[35] O’Brien KH. Social determinants of health: the how, who, and where screenings are occurring; a systematic review. Soc Work Health Care. 2019;58(8):719-745. doi:10.1080/00981389.2019.1645795 31431190

[36] Henrikson NB, Blasi PR, Dorsey CN, . Psychometric and pragmatic properties of social risk screening tools: a systematic review. Am J Prev Med. 2019;57(6)(suppl 1):S13-S24. doi:10.1016/j.amepre.2019.07.012 31753276

[37] Billioux A, Verlander K, Anthony S, Alley D. Standardized screening for health-related social needs in clinical settings: the Accountable Health Communities Screening Tool. National Academy of Medicine. May 30, 2017. Accessed November 3, 2023. https://nam.edu/standardized-screening-for-health-related-social-needs-in-clinical-settings-the-accountable-health-communities-screening-tool/

[38] Karran EL, G Cashin A, Barker T, ; Identifying Social Factors that Stratify Health Opportunities and Outcomes (ISSHOOs) Collaborative Core Research Group. The ‘*what’* and ‘*how*’ of screening for social needs in healthcare settings: a scoping review. Peer J. 2023;11:e15263. doi:10.7717/peerj.15263 37101795 PMC10124546

[39] Nohria R, Xiao N, Guardado R, . Implementing health related social needs screening in an outpatient clinic. J Prim Care Community Health. Published online August 17, 2022. doi:10.1177/21501319221118809 35978539 PMC9393584

