# Supplementary material for: Social Risk and Clinical Outcomes Among Adults With Type 2 Diabetes
Source: JAMA Netw Open. 2024 Aug 29;7(8):e2425996. doi: 10.1001/jamanetworkopen.2024.25996 (PMC11362860; doi:10.1001/jamanetworkopen.2024.25996)
Supplement: Supplement. — Data Sharing Statement [file jamanetwopen-e2425996-s001.pdf]

Data Sharing Statement

Walker. Social Risk and Clinical Outcomes Among Adults With Type 2 Diabetes. *JAMA Netw Open*. Published August 16, 2024. doi:10.1001/jamanetworkopen.2024.25996

Data
Data available: No

Additional Information
Explanation for why data not available: The data that support the findings of this study are available from the corresponding author, LEE, upon reasonable request
